# Predictive value of serum progesterone level on *β*-hCG check day in women with previous repeated miscarriages after *in vitro* fertilization

**DOI:** 10.1371/journal.pone.0181229

**Published:** 2017-07-14

**Authors:** Yong Jin Kim, Jung Ho Shin, Jun Yong Hur, Hoon Kim, Seung-Yup Ku, Chang Suk Suh

**Affiliations:** 1 Department of Obstetrics and Gynecology, Korea University College of Medicine, Seoul, South Korea; 2 Department of Obstetrics and Gynecology, Seoul National University Hospital, Seoul, South Korea; Michigan State University, UNITED STATES

## Abstract

**Objective:**

To evaluate the predictive value of the progesterone level at the beta-human chorionic gonadotropin (*β*-hCG) check day for ongoing pregnancy maintenance in *in vitro* fertilization (IVF) cycles in women with previous unexplained repeated miscarriages.

**Materials and methods:**

One hundred and forty-eight women, with visible gestational sac after IVF, were recruited in this observational study. All subjects had unexplained recurrent miscarriages in more than two previous IVF cycles. The progesterone level at the *β*-hCG check day (i.e. 14 days after oocyte retrieval) was assessed. The area under the curve (AUC) of the progesterone level was evaluated to predict the ongoing pregnancy or miscarriage outcomes.

**Results:**

The overall ongoing pregnancy rate was 60.8% (90/148). The cut-off value with *β*-hCG levels higher than 126.5 mIU/mL and with progesterone levels higher than 25.2 ng/mL could be the predictive factors for ongoing pregnancy maintenance (AUC = 0.788 and 0.826; sensitivity = 0.788 and 0.723; specificity = 0.689 and 0.833; *P* < 0.0001 and *P* < 0.0001, respectively). The miscarriage rates were 19.5% (15/77) in the women with *β*-hCG > 126.5 mIU/mL and 13.0% (10/77) in those with > 25.2 ng/mL. In the comparison of the ROC curves between both values, a similar significance was found. The subjects with *β*-hCG > 126.5 mIU/mL and progesterone > 25.2 ng/mL showed higher ongoing pregnancy rates [98.0% (49/50) vs. 41.8% (41/98)] than those with *β*-hCG ≤ 126.5 mIU/mL or progesterone ≤ 25.2 ng/mL.

**Conclusions:**

The progesterone level at 14 days after oocyte retrieval can be a good predictive marker for ongoing pregnancy maintenance in women with repeated IVF failure with miscarriage, together with the *β*-hCG level. The combined cut-off value of progesterone > 25.2 ng/mL and *β*-hCG > 126.5 mIU/mL may suggest a good prognosis.

## Introduction

The overall miscarriage rate in pregnant women after *in vitro* fertilization (IVF) was reported to be ~23% [1, 2]. For couples who conceive via IVF, miscarriage after confirmation of a visible intrauterine gestational sac (G-sac) can be another “hope torture” in a different way compared to infertility. Furthermore, unexplained recurrent miscarriages after IVF can be a challenging and frustrating condition for both patients and clinicians. Numerous previous studies have suggested the predictors for miscarriage after IVF, such as endometrial thickness [3] and pattern [4], anti-Müllerian hormone level [5], beta-human chorionic gonadotropin (*β*-hCG) [6], even previous miscarriage history [7]. However, these predictors may not be clinically valuable in terms of their sensitivity and specificity, especially at early pregnancy period, as the first checked serum *β*-hCG.

Progesterone is an essential hormone in the process of reproduction. It is involved in implantation and pregnancy maintenance. It has been used in IVF for luteal support after embryo transfer (ET) and early pregnancy maintenance in cases of positive *β*-hCG check [8]. It was suggested that progesterone plays a role in the modulation of maternal immune response [9], reduction of uterine contractility [10], and improvement of utero-placental circulation [11]. With the stimulation of hCG secreted by the placenta after implantation into the maternal side, progesterone is mainly produced by the corpus luteum in the early pregnancy period [12]. These premises implicate that not only the *β*-hCG level but also the maternal serum progesterone level in early pregnancy can be a predictor of maintenance of pregnancy. A recent report showed that progesterone levels on the *β*-hCG check day could be considered a predictor for heterotopic pregnancy after two or more embryos transferred during the IVF procedures [13]. However, there is limited knowledge on the predictive value of serum progesterone level in the early pregnancy period for miscarriage after implantation in the IVF cycles.

This study aimed to evaluate the predictive value of the progesterone level on the *β*-hCG check day for miscarriage after confirmation of a visible G-sac in women with miscarriage history who underwent IVF cycles.

## Materials and methods

### Study subjects

This prospective observational study was approved by our institutional review board with written informed consent (KUGH13147-005). The included subjects were women with visible G-sac in the uterine cavity at the 5^th^ week of gestation after *in vitro* fertilization (IVF) and with history of ≥ 2 unexplained miscarriages in the previous fresh or thawed embryo transfer cycles. The exclusion criteria were as follows: old age (> 42 years); obesity (BMI > 25 kg/m^2^); expected low response (AMH < 0.8 ng/mL); uterine and endometrial factor infertilities; presence of male factor infertility; metabolic, hepatic, and cardiovascular disorders; and multiple pregnancies.

### Controlled ovarian stimulation (COS) protocols

COS and IVF were performed as previously reported [14, 15]. For the GnRH agonist long protocol, the GnRH agonist triptorelin (Decapeptyl^®^, 0.1 mg/day; Ferring, Malmo, Sweden) was started in the mid-luteal phase of the previous cycle. After pituitary down-regulation, the triptorelin dose was reduced to 0.05 mg/day, and gonadotropin (Gonal-F^®^; Serono, Geneva, Switzerland) was added until either the leading follicle reached a mean diameter of 18 mm or two or more follicles reached a diameter of 17 mm. Treatment with 37.5–300 IU of gonadotropin, depending on the patients’ previous or anticipated responses, was initiated on the third day of the menstrual cycle. The treatment was then individualized and adjusted in accordance with the response. For the GnRH antagonist multiple dose flexible protocol, 37.5–300 IU of gonadotropin was started on the third menstrual cycle day. The GnRH antagonist cetrorelix (Cetrotide^®^, 0.25 mg; Serono, Geneva, Switzerland) was added daily, started when the leading follicle reached a diameter of 14 mm, and continued until either the leading follicle reached a mean diameter of 18 mm or two or more follicles reached a diameter of 17 mm. For both protocols, recombinant hCG (Ovidrel^®^, Serono, Geneva, Switzerland) was administered subcutaneously 36 hours before the ultrasonography-guided oocyte retrieval. The GnRH agonist or antagonist protocol was employed in accordance with the menstrual cycle day at the patient’s visit.

### *In vitro* fertilization (IVF)

The retrieved oocytes were cultured for 4 to 6 hours until insemination. Semen samples obtained via ejaculation on the morning of the oocyte retrieval day were liquefied at room temperature for 30 minutes and centrifuged using the SpermGrad (Vitrolife, Kungsbacka, Sweden) made of two gradients (45%/90%) at 1,500 rpm for 20 minutes. After removal of the supernatant, we layered 2 mL of the Universal IVF medium over the sperm pellet for centrifugation at 1,000 rpm for 10 minutes. After washing and conducting the swim-up procedure, only the sperm pellet in the supernatant was aspirated and used for the insemination. Fertilization was determined by the presence of 2 pronuclei (2PN) using an inverted microscope on the first day after insemination. The zygotes with 2PN were cultured individually in the microdrops of 25 μL of growth medium, G-1^™^ v5 (Vitrolife) overlaid with 8 mL of mineral oil (Sigma, USA) in Falcon 1007 culture dishes (Becton Dickinson Labware, Franklin Lakes, New Zealand) at 37°C under 6% CO_2_.

### Embryo transfer (ET), luteal support and pregnancy follow-up

ET was performed 3 days after oocyte retrieval. The embryos were graded according to their morphologies and cleavage rates; they were graded from I to V based on the number and uniformity of the blastomere and percentage of fragmentation. We defined good-quality embryos as those of morphologic grades I-II/V; embryos with blastomeres of equal size with no cytoplasmic fragments or with minor cytoplasmic fragments or blebs and up to two good-quality embryos were selected and transferred into the uterus.

The luteal phase was supported with daily 8% progesterone gel (Crinone^®^, Serono) initially for 14 days, starting on the day of oocyte retrieval. The serum *β*-hCG and progesterone levels were checked on the 14^th^ day after oocyte retrieval, and the G-sac was assessed via vaginal ultrasonography on the 21^st^ day after oocyte retrieval. If a G-sac was visualized, the gestational age was determined as the 5^th^ gestational week. The luteal support was continued until 8^th^ week of gestation. For surveillance of miscarriage, all subjects were followed up for up to 18 weeks of gestation.

### Statistical analysis

Data were analyzed using the Student’s t-test, Mann-Whitney U test, Chi-square test, Fisher’s exact test, and ROC curve, where appropriate. In all tests, significance was accepted for *P* values < .05. All data were analyzed using the Statistical Package for the Social Sciences for Windows (version 20.0, SPSS Inc.) and MedCalc (version 17.4.4, ScyMed).

## Results

### Baseline characteristics and overall pregnancy outcomes

In the present study, 148 women with positive *β*-hCG on the 14^th^ day after oocyte retrieval were enrolled. The basal characteristics in the present study are shown in Table 1. The overall ongoing pregnancy rate over 18 weeks of gestation was 60.8% (90/148), and the miscarriage rate was 39.2% (58/148). The mean *β*-hCG and progesterone levels at the 14^th^ day after oocyte retrieval were 157.3±79.5 mIU/mL and 29.9±17.4 ng/mL, respectively. There was a significant positive correlation between β-hCG and progesterone levels (rho = 0.352, *P*< .0001).

**Table 1:** Baseline characteristics of study subjects.

Variables	mean±S.D.
Number	148
Age (years)	
Wife	35.1±4.5
Husband	38.1±4.8
Cause of infertility	
Ovulatory	35.1% (52/148)
Tubal	31.1% (46/148)
Unexplained	33.8% (50/148)
No. of previous IVF cycles	2.5±0.8
Basal FSH (mIU/mL)	7.2±2.3
Basal AMH (ng/mL)	1.9±1.1
Protocols	
GnRH agonist	58.1% (86/148)
GnRH antagonist	41.9% (62/148)
No. of oocytes retrieved	9.7±5.7
No. of MII oocytes	6.7±4.4
No. of 2PN	4.9±3.8
Fertilization rate	73.1%
No. of embryos transferred	1.9±0.8
Pregnancy outcomes	
Ongoing pregnancy	60.8% (90/148)
Miscarriage	39.2% (58/148)
β-hCG (mIU/mL)	157.3±79.5
Progesterone (ng/mL)	29.9±17.4

### Comparison between the ongoing pregnancy maintenance and miscarriage groups

The comparison of the variables between the ongoing pregnancy maintenance and miscarriage groups is shown in Table 2. No significant difference was found in the age of the couples, cause of infertility, number of previous IVF cycles, basal FSH and AMH levels, GnRH agonist or antagonist protocol ratio, number of oocytes retrieved, and number of embryos transferred. The ongoing pregnancy maintenance group showed higher *β*-hCG and progesterone levels than the miscarriage group (196.1±82.6 mIU/mL vs. 137.5±45.6 mIU/mL, *P* < .0001; 37.5±16.7 ng/mL vs. 18.1±10.6 ng/mL, *P* < .0001).

**Table 2:** Comparison between ongoing pregnancy and miscarriage groups.

Variables	Ongoing pregnancy (n = 90)	Miscarriage (n = 58)
Age (years)		
Wife	34.8±4.9	35.5±3.8
Husband	37.8±4.6	38.2±5.7
Cause of infertility		
Ovulatory	35.6% (32/90)	34.5% (20/58)
Tubal	32.2% (29/90)	29.3% (17/58)
Unexplained	32.2% (29/90)	36.2% (21/58)
No. of previous IVF cycles	2.4±0.9	2.5±1.0
Basal FSH (mIU/mL)	7.2±2.6	7.2±3.1
Basal AMH (ng/mL)	1.9±1.5	1.8±0.8
Protocols		
GnRH agonist	57.8% (52/90)	58.6% (34/58)
GnRH antagonist	42.2% (38/90)	41.4% (24/58)
No. of oocytes retrieved	9.9±6.1	9.5±5.9
No. of MII oocytes	6.8±4.1	6.6±3.6
No. of 2PN	4.7±3.0	5.0±4.2
Fertilization rate	69.2%	75.8%
No. of embryos transferred	1.9±0.9	1.9±1.0
β-hCG (mIU/mL)	196.1±82.6^a^	137.5±45.6^a^
Progesterone (ng/mL)	37.5±16.7^b^	18.1±10.6^b^

^a^ and ^b^ *P*< .0001, *P*> .05 in the others

### ROC curve of the β-hCG and progesterone levels

**Fig 1:**
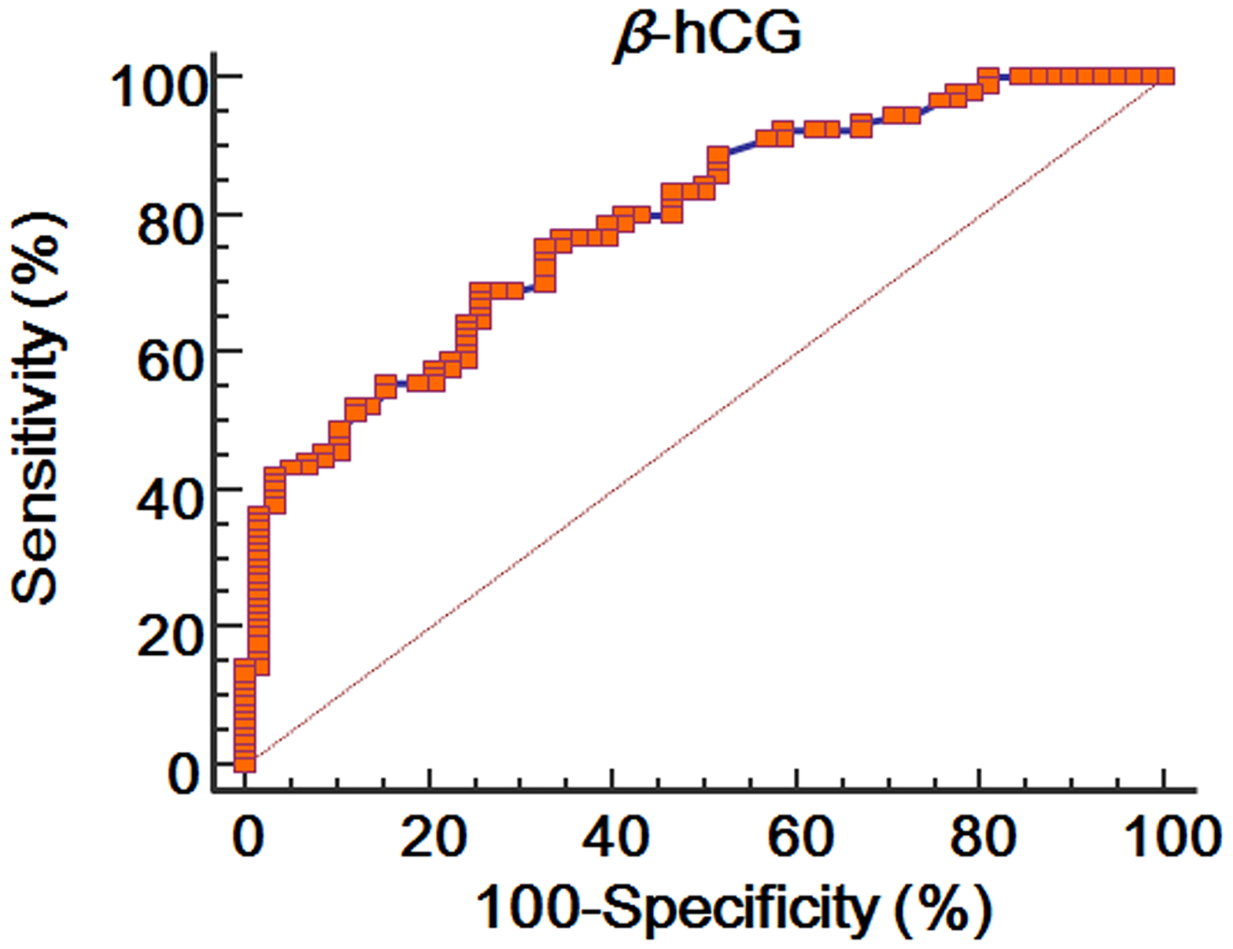
ROC curve of *β*-hCG level at 14 days after oocyte retrieval for predicting ongoing pregnancy.

**Fig 2:**
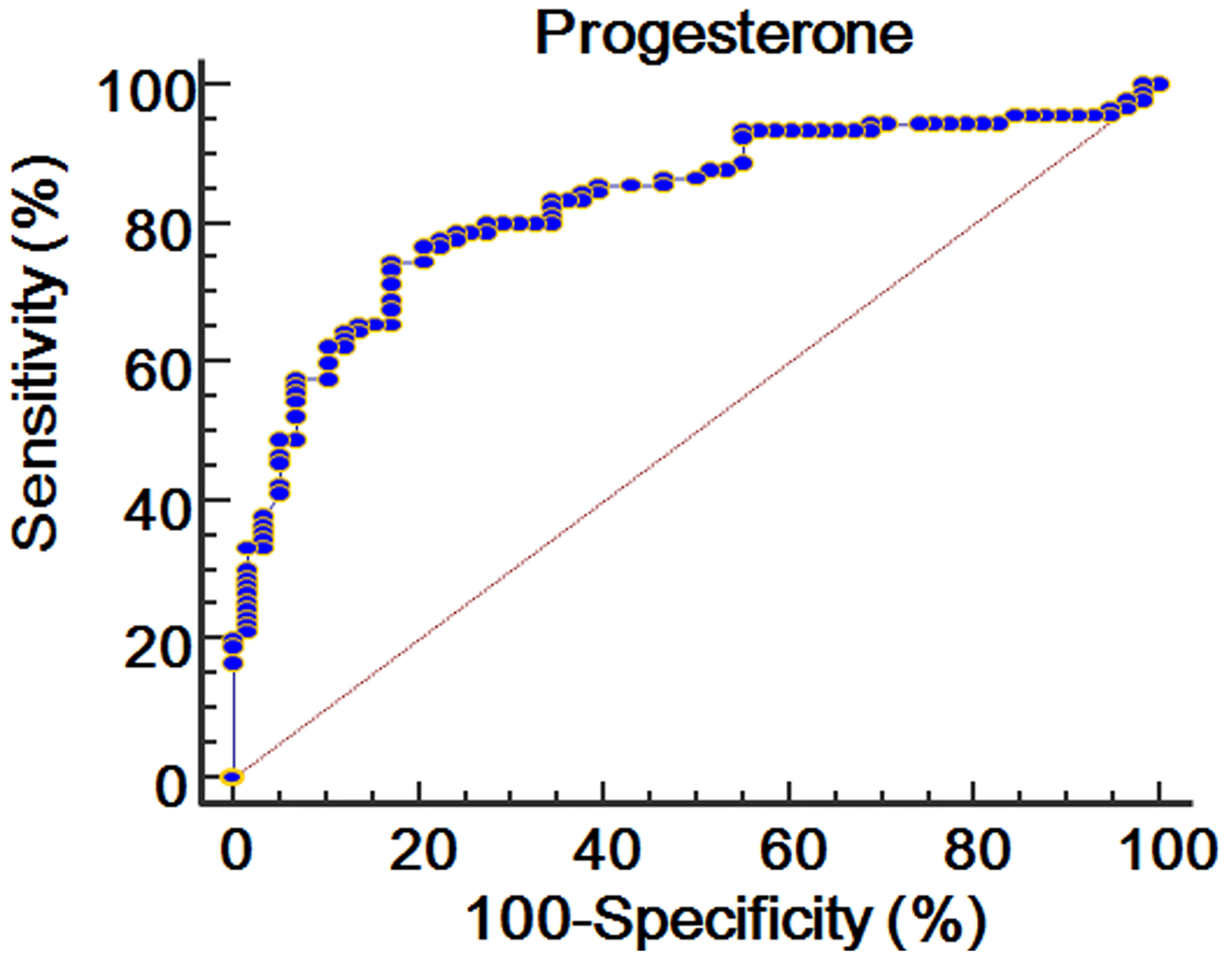
ROC curve of progesterone level at 14 days after oocyte retrieval for predicting ongoing pregnancy.

Figs 1 and 2 show the ROC curve of the *β*-hCG and progesterone levels for predictive maintenance of ongoing pregnancy. The area under the ROC curve (AUC) of the *β*-hCG level was 0.788 (standard error = 0.0369; 95% CI = 0.714 to 0.851; *P* < 0.0001), and its cut-off value was higher than 126.5 mIU/mL (sensitivity = 68.9%; specificity = 74.1%). The AUC of the progesterone level was 0.826 (standard error = 0.0343; 95% CI = 0.755 to 0.883; *P* < 0.0001), and its cut-off value was higher than 25.2 ng/mL (sensitivity = 74.4%; specificity = 82.8%).

### Comparison using the cut-off values of the β-hCG and progesterone levels

Using the cut-off value of the *β*-hCG level, the subjects with *β*-hCG > 126.5 mIU/mL showed higher ongoing pregnancy rates [80.5% (62/77) vs. 39.4% (28/71), *P* < .0001] and lower miscarriage rates [19.5% (15/77) vs. 60.6% (43/71), *P* < .0001] than those with *β*-hCG ≤ 126.5 mIU/mL, without a difference in the other variables (Table 3).

**Table 3:** Comparison of ongoing pregnancy outcomes according to cut-off value of *β*-hCG (> 126.5 ng/mL) at 14 days after oocyte retrieval.

Variables	*β*-hCG > 126.5 mIU/mL (n = 77)	*β*-hCG ≤ 126.5 mIU/mL (n = 71)
Age (years)		
Wife	34.3±4.6	35.6±5.4
Husband	38.1±5.1	40.1±4.1
Cause of infertility		
Ovulatory	35.1% (27/77)	35.2% (25/71)
Tubal	33.8% (26/77)	28.2% (20/71)
Unexplained	31.2% (24/77)	36.6% (26/71)
No. of previous IVF cycles	2.5±1.2	2.6±1.0
Basal FSH (mIU/mL)	8.0±3.0	8.6±4.8
Basal AMH (ng/mL)	2.0±1.2	1.8±0.6
Protocols		
GnRH agonist	57.1% (44/77)	59.2% (42/71)
GnRH antagonist	42.9% (33/77)	40.8% (29/71)
No. of oocytes retrieved	10.1±5.7	9.7±7.4
No. of MII oocytes	6.7±4.7	6.8±5.3
No. of 2PN	4.9±3.2	4.8±3.9
Fertilization rate	73.2%	70.6%
No. of embryos transferred	1.8±1.0	1.6±0.8
Pregnancy outcomes		
Ongoing pregnancy	80.5% (62/77)^a^	39.4% (28/71)^a^
Miscarriage	19.5% (15/77)^a^	60.6% (43/71)^a^

^a^ *P*< .0001

Using the cut-off value of the progesterone level, the subjects with progesterone > 25.2 ng/mL showed higher ongoing pregnancy rates [87.0% (67/77) vs. 32.4% (23/71), *P* < .0001] and lower miscarriage rates [13.0% (10/77) vs. 67.6% (48/71), *P* < .0001] than those with progesterone ≤ 25.2 ng/mL, without a difference in the other variables (Table 4).

**Table 4:** Comparison of ongoing pregnancy outcomes according to cut-off value of progesterone (> 25.2 ng/mL) at 14 days after oocyte retrieval.

Variables	Progesterone > 25.2 ng/mL (n = 77)	Progesterone ≤ 25.2 ng/mL (n = 71)
Age (years)		
Wife	34.6±4.8	35.3±5.2
Husband	38.9±5.3	39.8±3.9
Cause of infertility		
Ovulatory	33.8% (26/77)	36.6% (26/71)
Tubal	32.5% (25/77)	29.6% (21/71)
Unexplained	33.8% (26/77)	33.8% (24/71)
No. of previous IVF cycles	2.4±1.8	2.7±0.3
Basal FSH (mIU/mL)	7.7±4.2	8.1±2.4
Basal AMH (ng/mL)	1.9±1.3	2.0±0.6
Protocols		
GnRH agonist	59.7% (46/77)	62.0% (44/71)
GnRH antagonist	40.3% (31/77)	38.0% (27/71)
No. of oocytes retrieved	9.9±6.1	10.0±6.5
No. of MII oocytes	6.8±4.6	7.1±5.1
No. of 2PN	4.8±3.1	5.1±3.5
Fertilization rate	70.6%	71.8%
No. of embryos transferred	1.9±0.8	1.9±0.9
Pregnancy outcomes		
Ongoing pregnancy	87.0% (67/77)^a^	32.4% (23/71)^a^
Miscarriage	13.0% (10/77)^a^	67.6% (48/71)^a^

^a^*P*< .0001

**Fig 3:**
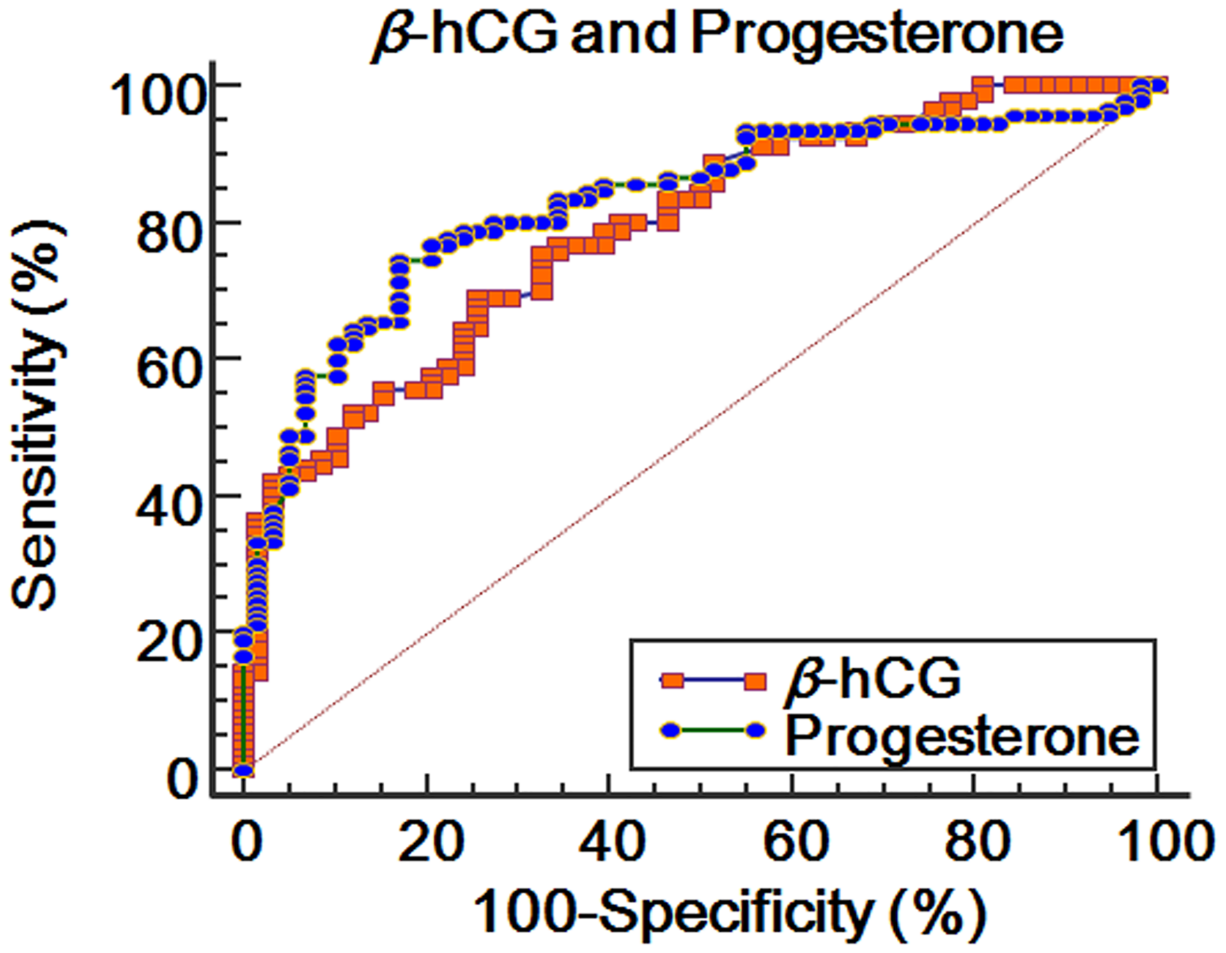
Comparison of ROC curves between of *β*-hCG and progesterone levels at 14 days after oocyte retrieval for predicting ongoing pregnancy.

In the comparison between the subjects with *β*-hCG > 126.5 mIU/mL and those with progesterone > 25.2 ng/mL, there was no significant difference in the variables (Table 5). In the comparison of the ROC curves between both values, a similar significance was found (difference between the areas = 0.0378; standard error = 0.0500; 95% CI = -0.0602 to 0.136; *P* = 0.4594) (Fig 3).

**Table 5:** Comparison of pregnancy outcomes between IVF patients with *β*-hCG > 126.5 mIU/mL and progesterone > 25.2 ng/mL at 14 days after oocyte retrieval.

Variables	*β*-hCG > 126.5 mIU/mL (n = 77)	Progesterone > 25.2 ng/mL (n = 77)
Age (years)		
Wife	34.3±4.6	34.6±4.8
Husband	38.1±5.1	38.9±5.3
Cause of infertility		
Ovulatory	35.1% (27/77)	33.8% (26/77)
Tubal	33.8% (26/77)	32.5% (25/77)
Unexplained	31.2% (24/77)	33.8% (26/77)
No. of previous IVF cycles	2.5±1.2	2.4±1.8
Basal FSH (mIU/mL)	8.0±3.0	7.7±4.2
Basal AMH (ng/mL)	2.0±1.2	1.9±1.3
Protocols		
GnRH agonist	57.1% (44/77)	59.7% (46/77)
GnRH antagonist	42.9% (33/77)	40.3% (31/77)
No. of oocytes retrieved	10.1±5.7	9.9±6.1
No. of MII oocytes	6.7±4.7	6.8±4.6
No. of 2PN	4.9±3.2	4.8±3.1
Fertilization rate	73.2%	70.6%
No. of embryos transferred	1.8±1.0	1.9±0.8
Pregnancy outcomes		
Ongoing pregnancy	80.5% (62/77)	87.0% (67/77)
Miscarriage	19.5% (15/77)	13.0% (10/77)

*P*> .05 in all variables

### Significance of the combined cut-off values of the β-hCG and progesterone levels

Table 6 shows the comparison of the ongoing pregnancy outcomes according to the combined cut-off values of the *β*-hCG and progesterone levels. The subjects with *β*-hCG > 126.5 mIU/mL and progesterone > 25.2 ng/mL showed higher ongoing pregnancy rates [98.0% (49/50) vs. 41.8% (41/98)] than those with *β*-hCG ≤ 126.5 mIU/mL or progesterone ≤ 25.2 ng/mL.

**Table 6:** Comparison of pregnancy maintenance outcomes according to combined cut-off value of *β*-hCG (> 126.5 mIU/mL) and progesterone (> 25.2 ng/mL) at 14 days after oocyte retrieval.

	*β*-hCG > 126.5 mIU/mL and progesterone > 25.2 ng/mL (n = 50)	*β*-hCG ≤ 126.5 mIU/mL or progesterone ≤ 25.2 ng/mL (n = 98)
Ongoing pregnancy	98.0% (49/50)^a^	41.8% (41/98)^a^
Miscarriage	2.0% (1/50)^a^	58.2% (57/98)^a^

*P*< .0001 by Fisher’s exact test

## Discussion

### Study subjects and their overall pregnancy outcomes

The *β*-hCG level could be a predictive factor for ongoing pregnancy in IVF cycles. However, the specificity using only the *β*-hCG level was not sufficient for predicting ongoing pregnancy. The present study aimed to evaluate the predictive value of the progesterone level at the *β*-hCG check day (15 days after oocyte retrieval) for miscarriage in women with unexplained repeated miscarriages in previous IVF cycles and with visible G-sacs. Based on our results, the progesterone level combined with the *β*-hCG level can be suggested as a valuable predictor for ongoing pregnancy maintenance over 18 weeks of gestational age in women with visible G-sac after IVF cycles and with unexplained repeated miscarriages in previous IVF cycles.

In the overall pregnancy outcomes, the ongoing pregnancy maintenance rate over 18 weeks was 60.8% in the subjects with unexplained repeated miscarriages in previous IVF cycles, which was lower than the overall ongoing pregnancy maintenance rate in the whole group of patients with visible G-sac in our center (85.5%); such a finding is consistent with those of previous reports [1, 2]. The subjects showed a mean *β*-hCG level of 157.3±79.5 mIU/mL and a mean progesterone level of 29.9±17.4 ng/mL 14 days after oocyte retrieval, which is similar with those of previous reports [16–18]. In the comparison of the *β*-hCG and progesterone levels, significantly higher levels were shown in the ongoing pregnancy maintenance group than in the miscarriage group, which is also consistent with those of previous studies [16–18]. A previous study revealed that patients with repeated miscarriages showed significantly decreased hCG and progesterone secretion compared to healthy controls [19].

### Cut-off values of the β-hCG and progesterone levels

Maternal serum progesterone level may indicate the corpus luteal responsiveness to hCG secreted from the placenta in the early pregnancy period. Owing to the importance of progesterone support in the early pregnancy period, maintenance of pregnancy may depend on the production of progesterone from the corpus luteum in spite of exogenous luteal support. In the ROC curve analysis, not only the serum *β*-hCG level but also the progesterone level was found to have a good predictive value for ongoing pregnancy maintenance or miscarriage. Although without a significant difference, the cut-off value > 25.2 ng/mL of the progesterone level at 14 days after oocyte retrieval showed a higher AUC, sensitivity, and specificity than the cut-off value > 126.5 mIU/mL of the *β*-hCG level for predicting ongoing pregnancy maintenance or miscarriage. While the *β*-hCG level could predict 80.5% of ongoing pregnancy maintenance up to 18 weeks of gestational age using the cut-off value > 126.5 mIU/mL, the progesterone level could predict 87.0% of ongoing pregnancy maintenance using the cut-off value > 25.2 ng/mL. As shown in Table 6, the subjects with combined cut-off values *β*-hCG > 126.5 mIU/mL and progesterone > 25.2 ng/mL showed a 98.0% ongoing pregnancy maintenance rate.

**Fig 4:**
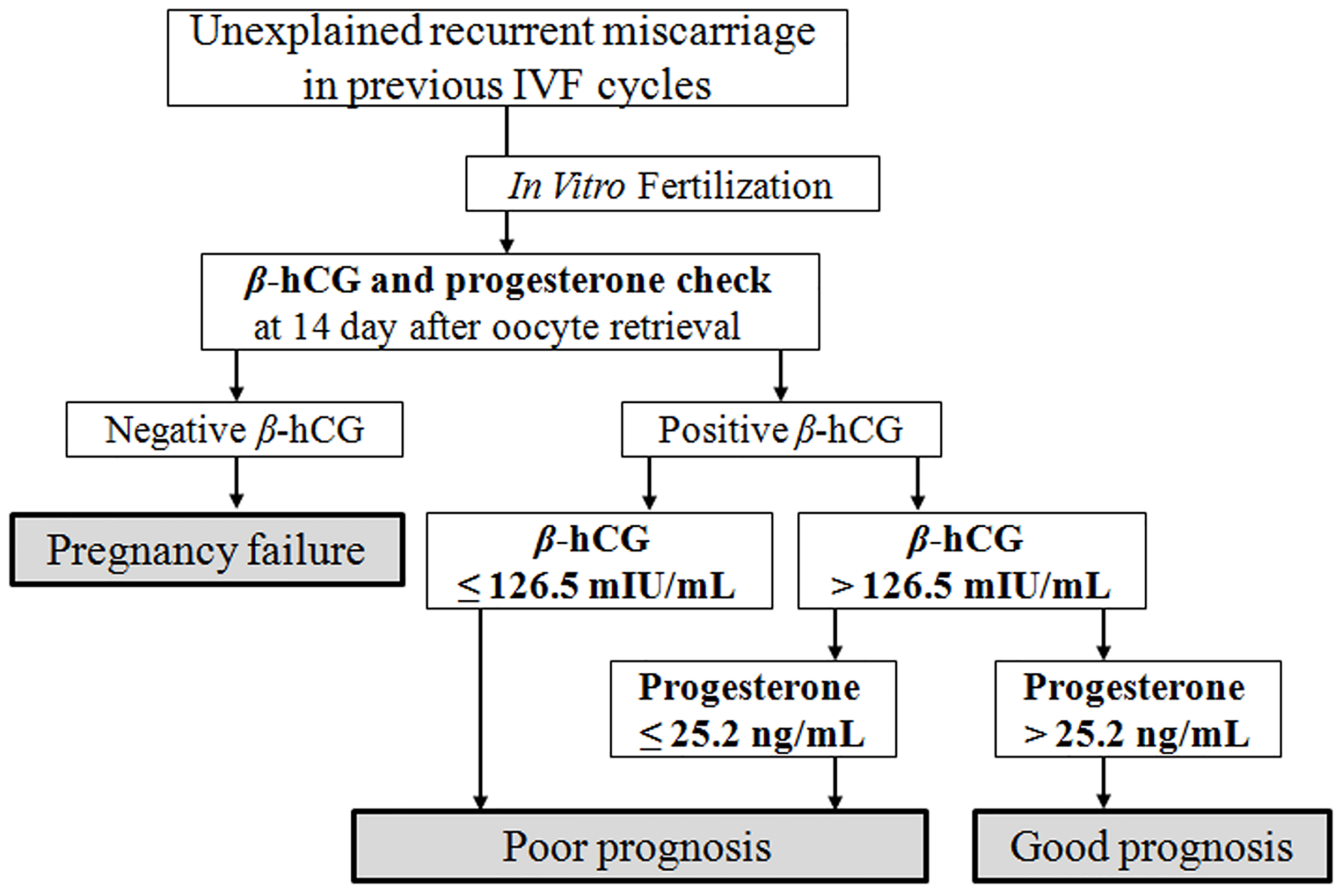
Suggested predictive value of *β*-hCG and progesterone levels during the follow-up of pregnant IVF cases with previous unexplained recurrent miscarriages.

To date, few studies reported the progesterone level at the *β*-hCG check day as a predictive marker for ongoing pregnancy maintenance. A recent study, which used earlier *β*-hCG serum samples than in the present study (obtained 7 days after D3 ET), suggested a predictive value of ongoing pregnancy maintenance consistent with our results [20]. Another report showed that serum progesterone levels > 17 ng/mL at 7 days after ET could be a good predictor of IVF cycle outcomes via an ROC analysis [21]. Our present results suggest that the progesterone level at 14 days after oocyte retrieval might be a good prognostic marker for ongoing pregnancy maintenance and could be more useful when combined with the *β*-hCG level. We suggest a clinical strategy for repeated miscarriages after IVF as shown in Fig 4. Although the management for poor prognosis has been a matter of debate, particularly in repeated miscarriages after IVF, some reports suggested increasing the dose of luteal progesterone support. Although not restricted to IVF cycles, a recent meta-analysis suggested that supplementation with progestogens may reduce the incidence of recurrent miscarriages and seems to be safe for the fetuses in women with a history of recurrent miscarriage [22].

### Points to consider for interpretation of present data

First, the results of the present study were retrieved from the subjects who underwent IVF cycles using the ovarian gonadotropin stimulation protocol, which was different from the general pregnancy cases of unstimulated ovaries and corpus luteum. A previous study revealed that gonadotropin promotes progesterone synthesis and output from the granulosa cells of the ovaries without luteinization in gonadotropin-stimulated IVF cycles, which was different from the natural cycle [23]. Second, the subjects in this study were unique in terms of their repeated miscarriages after pregnancy in the previous IVF cycles. Thus, there may be concerns in the generalization of these results to all IVF cycles, particularly when applied to the first IVF cycle. However, this study is the first study on the progesterone level at the *β*-hCG check day in IVF patients with previous repeated miscarriages. Lastly, the cost-effectiveness with additional progesterone samples at the *β*-hCG check day in IVF procedures should be analyzed based on the hormonal control of endometrium [24] in the future.

## Conclusions

Our study showed that the progesterone level 14 days after oocyte retrieval can be a good predictive marker for ongoing pregnancy maintenance in women with repeated IVF failure, together with the *β*-hCG level. The combined cut-off value of progesterone > 25.2 ng/mL and *β*-hCG > 126.5 mIU/mL may suggest a good prognosis.
